# Psychometric properties of the generalized anxiety disorder-7 (GAD-7) in a sample of workers

**DOI:** 10.3389/fpsyt.2023.999242

**Published:** 2023-03-27

**Authors:** César Merino-Soto, Marisol Angulo-Ramos, Lillian V. Rovira-Millán, Ernesto Rosario-Hernández

**Affiliations:** ^1^Instituto de Investigación de Psicología, Universidad de San Martín de Porres, Lima, Perú; ^2^Sociedad Científica Peruana de Enfermería Pediátrica, Lima, Peru; ^3^Psychology Program, Social Sciences Department, University of Puerto Rico, Cayey, Puerto Rico; ^4^Clinical Psychology Programs, School of Behavioral and Brain Sciences, Ponce Health Sciences University, Ponce, Puerto Rico; ^5^Ponce Research Institute, Ponce Health Sciences University, Ponce, Puerto Rico

**Keywords:** differential item functioning, GAD-7, item response theory, psychometric properties, validity, anxiety, workers

## Abstract

**Objective:**

To evaluate the psychometric properties of the GAD-7 by obtaining evidence of internal structure (dimensionality, precision and differential functioning of items) and association with external variables.

**Methods:**

A total of 2,219 protocols from three different studies conducted with Puerto Rican employees that administered the GAD-7 were selected for the current study. Item response theory modeling was used to assess internal structure, and linear association with external variables.

**Results:**

The items were adapted to a graduated response model, with high similarity in the discrimination and location parameters, as well as in the precision at the level of the items and in the total score. No violation of local independence and differential item functioning was detected. The association with convergent (work-related rumination) and divergent (work engagement, sex, and age) variables were theoretically consistent.

**Conclusion:**

The GAD-7 is a psychometrically robust tool for detecting individual variability in symptoms of anxiety in workers.

## Introduction

According to Canino et al. (1), the prevalence of generalized anxiety in the population of Puerto Rico is 5.2%, which occupies a third place after depression (9.7%) and social phobia (6.3%). On the other hand, the most recent statistics available from the Department of Health of Puerto Rico shows that 53.8% of their patients have been diagnosed with generalized anxiety disorder (2). Rodríguez-Kierce (3) note that Puerto Rico’s healthcare system still experiences persistent budget problems, hospital financial difficulties, and a staffing shortfall of healthcare workers. Moreover, Rodríguez-Kierce add that when not directly flying beyond the island to receive care those patients that can, patients frequently must wait months to see a healthcare provider. Nevertheless, psychologists and other related health professionals, particularly physicians, are the primary point of contact for persons who may need health intervention services (4). They are also exposed to provide a professional perspective on mental health in primary health care, and one of the mental health explorations is likely to uncover psychological vital signs (5). Depression, anxiety, and anger symptoms are all covered in this category. The subject of the current instrumental study is anxiety, and it has been established that evaluating it in primary health care is important for early therapies (4). However, anxiety can be examined in a variety of settings, including the workplace, where anxiety disorders are common.

Anxiety disorders in workers are linked to psychosocial factors at work (6), such as poor protection strategies, a lack of social support (7), and the uncertainty of the future job (8), and labor demands related to expected work efficiency ((9, 10)). These requirements might be defined by single or several components of the job, such as precision, speed, and the ability to complete difficult tasks (11). When looking at the relationship between psychosocial work elements and adaptive or maladaptive reactions, it is clear that job demands are one of the most common causes of increased stress and anxiety (6, 9). Additionally, findings from some research in Puerto Rico suggest that psychosocial factors such as work demands, job insecurity and even boredom at work are associated with the manifestation of anxiety symptoms (12–14). Furthermore, evaluating employee psychological distress and screening them for mental disorders has a considerable potential to be advantageous to both the employees and the organizations through early detection, connection to treatment, increased worker productivity, and lower health insurance costs.

In research on occupational exposures and their psychological and medical effects on workers, anxiety measurements with general symptom scales [e.g., BAI: (15); GHQ- 28: (16); STAI: (17)], it is common practice (e.g., (18)). But, the development of brief and inexpensive measures is handy in response to the requirement to assess anxiety symptoms in primary health care, particularly in the workplace, and a unidimensional measure may be a promising choice (19). The GAD-7 is strongly advised for primary health care (20) because is one of the most extensively used instruments in the world, as well as one of the most validated (21). Studies (e.g., (22)) have highlighted the GAD-7’s sensitivity and specificity in detecting anxiety disorders, an approach that has been included into the criteria validity or association with other factors (23). This evidence of validity is important to choose the GAD-7 in professional practice because of the usage provided to it, but it is not the only evidence of validity that is required. Furthermore, evidence of validity based on associations with other variables is dependent on the instrument’s goal, but *a priori* requires that the instrument’s internal structure be as expected theoretically and invariant across groups. Dimensionality, measurement invariance, and dependability are all factors to consider while evaluating the internal structure. As a result, in order to progress in the evaluation of additional sources of validity, such as the association with criteria, this proof of validity must be developed with *a priori* conditions. However, while the GAD-7’s validation studies imply that its qualities are unchanging, these validations are focused on non-Hispanic populations, creating a study space focused on psychometric properties in Hispanic populations.

Although there are several psychometric studies around world [e.g., (24–34)], and at least 16 validation studies there is focus in various languages (including Spanish-speaking), in various specific samples and contexts (35) on the GAD-7, one cannot infer the validity of an instrument in a certain population by extracting validity information from other studies, for example from different cultures (36, 37), because this does not guarantee that the induced parameters are exactly preserved. Therefore, it is imperative to examine the psychometric properties of the GAD-7 in samples other than the clinical and general population, since the GAD-7 is being frequently used in the work context in Puerto Rico (e.g., (12, 13, 38)).

However, none of the studies reviewed by Bártolo et al. (35) nor the one carried out by Pagán-Torres et al. (39) in Puerto Rico, had employee samples; thus, indicating an apparent research gap that needs to be addressed. Although a recent study validated the GAD-7 in general practitioners, even though the participants were dispersed across multiple private and public hospitals, but included only one occupation (40). In this sense, it is unknown whether factors such as more diverse group of employees in different occupations, the type of job (permanent/temporary), position (managerial/non-managerial) or type of organization (public/private) can be influential variables in the variability of the psychometric properties of the GAD-7, precisely because these studies are relatively absent. This psychometric variability can occur in parameters that define the internal structure of a measure, such as the magnitude of factor loadings (i.e., significance of items on the latent construct), in intercepts (i.e., differences in average responses on the construct scale), or in the number of factors (i.e., additional dimensions).

The use of the GAD-7 was intended to describe and screen for anxiety not only in clinical groups or samples in healthcare, but also in the general population. This is a general context, of non-specific grouping regarding the activity of the evaluated, where the evaluated are not naturally gathered for long periods of time, or forming a homogeneous group. In contrast, in a context where workers are naturally included, such as their work environment, and for long periods of time, the evaluation of generalized anxiety can have a preventive role in general mental health, specifically for the early detection of generalized anxiety, and in the distinction and degree of covariation with work-related anxiety. And, with validated measures of anxiety, like GAD-7, the services that the work institution provides to its workers can also provide the prevention of mental health, through the early evaluation of anxiety symptoms. Through qualified personnel to apply a screening, this detection can involve the GAD-7 for referral to external services, or to intramural services.

The earlier gap is added to the apparent inconsistency in some validation studies of the internal structure of the GAD-7, regarding the misspecification of the number of dimensions [e.g., more than one dimension (41, 42)], or the existence of significant correlated errors [e.g., (35, 43)]. This inconsistency has been highlighted to motivate validation studies to avoid inducing its validity and corroborate its dimensionality [e.g., (40)]. Also, meanwhile the unidimensionality remains almost intact in internal structure studies, there are other aspects that show less consistency. In this sense, although it has been recognized that the sensitivity and specificity of GAD-7 are acceptable at certain cut-off points, more validity studies are still needed (44), and many of these appear to require invariance studies, appropriate estimates for ordinal categorical variables (i.e., GAD-7 items), as well as a more thorough evaluation with other measurement models, such as item response theory (IRT).

The graded response model (grm; (45)) and the generalized partial credit model (46) are two IRT models with rising use in social sciences in general and health sciences in particular (47). Other polytomous item models coexist (e.g., rating scale model: (48); partial credit model: (49)), but they impose limits that are unlikely to be adequate for representing GAD-7 responses as the equality of the discrimination parameter in the items of the instrument, the same distance between the response thresholds and a combination of both restrictions. Table 1 of Nguyen et al. (50) provides a good summary of these models, as well as a full discussion in other sources (e.g., (51)). Both are employed in polytomous responses, which have a number of response possibilities more than two and an ordinal scale of the responses to the items.

**Table 1:** Sociodemographic characteristics of the sample.

Variable	*N*	%		Association test and effect size
Samples				*χ*^2^ (df: 2) = 105.74 ***
Sample 1 (n_1_)	898	40.5		
Sample 2 (n_2_)	518	23.3		
Sample 3 (n_2_)	803	36.2		
Total	2,219	100.0		
				
	n_1_	n_2_	n_3_	
Sex (*n* = 2,118)				*χ*^2^ (df: 2) = 7.17*, V = 0.058
Males	403	187	320	
Females	469	291	448	
				
Marital status (n = 2,198)				*χ*^2^ (df: 8) = 10.74, V = 0.07
Single	297	164	234	
Married	378	241	369	
Widowed	29	8	19	
Divorced	103	48	80	
Living together	88	50	90	
				
Job position (*n* = 2,159)				*χ*^2^ (df = 2) = 8.41*, V = 0.06
Managerial	207	92	199	
Non-Managerial	679	403	579	
				
Tenure (*n* = 2,173)				*χ*^2^ (df = 2) = 4.55, V = 0.046
Permanent	683	395	642	
Temporary	199	109	145	
				
Organization type (*n* = 2,185)				*χ*^2^ (df = 4) = 46.71***, V = 0.146
Public–State	288	158	209	
Public–Federal	88	17	33	
Private	511	326	555	
				
Work shift (*n* = 1,693)				*χ*^2^ (df = 3) = 4.06, V = 0.049
Day	629	–	589	
Evening	55	–	51	
Night	21	–	23	
Rotating	187	–	138	

*p < 0.05, **p < 0.01, ***p < 0.01; V = Cramer V coefficient.

To date, classical test theory has been used to investigate the psychometric aspects of the GAD-7, while item response theory (IRT) has been used less frequently to extract parameters from the GAD-7 items. Compared to classical test theory, IRT modeling has several distinct advantages, such as the modeling of item-latent variable relationships (θ) with nonlinear functions, item parameters in the latent attribute metric, and conditional precision parameters ((47, 50–52)). Despite the fact that there appears to be an increase in GAD-7 validation research around the world, there are still some elements that need to be examined because they were not addressed in those studies. Other recent research, except for a study among community adults in Germany (53), do not conduct measurement invariance analyses (e.g., (10, 24, 54)). Similarly, a recent Latin American study (25) confirmed the unidimensionality and good validity of the items with the latent construct, partially utilizing categorical variable technique, but did not disclose measurement invariance or the evaluation of any residual covariations. This implies that there are still unanswered questions about the GAD-7’s measurement quality.

The Bayesian graded-response IRT model was successfully implemented in a few additional recent GAD-7 assessments, but IRT models were not compared (28). Another IRT framework study only tested the graded response model (33), and both studies raise the question of whether different IRT models are adequate for the data gathered with GAD-7, particularly whether they are more efficient and require fewer parameters.

Recently, there is only one validity of the GAD-7 in Puerto Rico (39), in an apparent general sample of adults (n = 302). Although it is reasonable for this sample to include workers, it could also include adults without temporary work activity. This study validated the unidimensionality, also obtaining high reliability of the score. However, other qualities such as those obtained with IRT modeling were not reported. In this sense, the present validity study does not mean inducing the properties of the GAD-7 from the first Puerto Rican study (39), but rather evaluating the psychometric properties of the GAD-7 with new tools. In this way, the validation of the GAD-7 in an infrequently represented population (i.e., a labor group in its own workplace, with relative homogeneity) avoids inducing validity evidence obtained from different samples in the studies (36, 37).

Since the GAD-7 is being used in workplace contexts, it is necessary to examine whether its psychometric properties are equally good as with clinical and general samples; and therefore, thus we do not fall into inducing the validity of their scores from other culturally and contextually different samples. Given the foregoing, the purpose of the current study was to examine the psychometric properties of the GAD-7 in a sample of workers. In addition, we used IRT modeling to assess the psychometric features of the GAD-7 in a sample of Puerto Rican employees. Because there have been no previous investigations in Puerto Rico on the validity of the GAD-7, focused with IRT modeling in data from grouped workers, the current study adds to instrument research in this country, as well as international research on the psychometric properties of the GAD-7 in general. IRT modeling was used to determine the IRT model that best fit the distribution of responses, as well as to get item parameters and the interaction with other variables relevant to the work environment, especially since the study sample was centered on workers.

## Materials and methods

### Participants

The sample used in the current study were secondary data obtained from workers of a variety of work organizations, of three researches conducted by the last two authors in Puerto Rico. A total of 2,219 protocols from three different research conducted by the authors (12, 13, 55) in Puerto Rico and each selected through a non-probabilistic sample and distributed into these three large groups: sample 1 (n = 898, 40.5%), sample 2 (n = 518, 253.3%), and sample 3 (n = 803, 36.2%). The characteristic of the whole sample such as sex, job position, among other variables, are shown in Table 2. The sample was composed of 54.4% of females, 22.4% occupied a managerial position, 62.7% of the employees worked for the private sector.

**Table 2:** Number of latent dimensions: EKC and AISP criterions.

# factors	Empirical Kaiser Criterion (EKC)		Automated item selection procedure (AISP)		
	Sample	Reference	0.40	0.50	0.60
1	4.97	1.11	1	1	1
2	0.45	0.37	1	1	1
3	0.41	0.35	1	1	1
4	0.39	0.32	1	1	1
5	0.27	0.28	1	1	1
6	0.27	0.27	1	1	1
7	0.21	0.23	1	1	1

### Measures

#### Anxiety

To measure anxiety, we used the GAD-7 (19), which was translated and validated into Spanish by García-Campayo et al. (27). The GAD-7 is a seven-item self-report questionnaire that measures general anxiety symptomatology and asked patients how often, during the last 2 weeks, they were bothered by each symptom. Response options are “not at all,” “several days,” “more than half the days,” and “nearly every day,” scored as 0, 1, 2, and 3, respectively. In addition, an item to assess duration of anxiety symptoms was included. When originally developing the GAD-7, Spitzer et al. (19) selected 9-items that reflected the symptom criteria of general anxiety disorder of the DSM-IV, but finally they selected the best correlated 7-items. Based on an assumed unidimensionality, the GAD-7 is interpreted as the sum of the responses to all items. Authors of the scale reported a Cronbach’s alpha coefficient of 0.93. In terms of its construct validity, internal structure was supported by factor analysis technique and convergent validity with its association to similar measures such as the Beck Anxiety Inventory and the anxiety subscale of the Symptom Checklist-90. An item example is: “Feeling nervous, anxious, or on edge.” We used the Spanish version for Puerto Rico, available at https://www.phqscreeners.com/select-screener.

#### Work engagement

We used the Utrech Work Engagement Scale - 3 items (UWES-3; (56)), which is an ultra-abbreviated measure of work engagement constructed with three items (24, 52, 57) from the original UWES-17 (58), one from each of the subscale: Vigor (“*At my work, I feel bursting with energy*”), Dedication (“*I am enthusiastic about my job*”) and Absorption (“*I am immersed in my work*”). Spanish-speaking studies have supported its psychometric properties (Calderón-De (59, 60)). In the current study, the unidimensionality of the UWES-3 was supported by a CFA analysis using the method of ULSMV, *χ^2^* = 0.00 (0), CFI = 1.000, uSRMR = 0.038 (90% CI = 0.001; 0.037), RMSEA = 0.093[90% CI = 0.085; 0.101]; reliability of the UWES-3 using the omega (*ω*) was 0.821 (95% CI = 0.799; 0.841).

#### Rumination

To measure work-related rumination, we used the Work-Related Rumination Scale (WRRS), which was originally developed by Cropley et al. (61) and has 15 questions using a 5-point Likert scale (1 = very seldom or never, 2 = seldom, 3 = sometimes, 4 = often, and 5 = very often or always). According to Cropley et al., results using the factor analytic technique support a three-factor internal structure of the WRRS, which are affective rumination, problem-solving pondering, and detachment; and authors reported their reliability *via* Cronbach’s alpha of 0.90, 0.81, and 0.88, respectively. An item example is: “Do you become tense when you think about work-related issues during your free time? In the current study, we used the WRRS-Spanish Version (WRRS-SV; (38)) validated with a sample of Puerto Rican workers in which were retained 11 of the 15 original items; therefore, we used items 1, 7, 9, and 15 of the affective rumination subscale; 2, 4, 8, and 11 of the problem-solving pondering subscale; and 3, 10, and 12 of the detachment subscale that now composed the WRRS-SV. In the current study, a three-factor of the WRRS-SV was supported by a CFA analysis using the method of ULSMV, *χ^2^* = 608.395 (62), CFI = 0.950, uSRMR = 0.038 [90% CI = 0.037; 0.039], RMSEA = 0.079 [90% CI = 0.074; 0.085]; reliability of subscales of affective rumination, problem-solving pondering, and detachment of the WRRS-SV using the omega was *ω = 0*.863 (95% CI = 0.851; 0.874), *ω = 0*.740 (95% CI = 0.720; 0.760), and *ω = 0*.714 (95% CI = 0.688; 0.738), respectively. An item example is: “Do you become tense when you think about work-related issues during your free time?

#### Social desirability

We used the Social Desirability Scale developed by Rosario-Hernández and Rovira Millán (63). This is a 11-item instrument in a Likert-agreement response format ranging from 1 (Totally Disagree) to 6 (Totally Agree), which pretend to measure a response bias in which people respond to a test thinking what is acceptable socially. Authors report its internal consistency through Cronbach’s alpha to be 0.86, which is an excellent reliability coefficient. Factor analysis results suggest that the Social Desirability Scale internal structure has only one factor. As part of the current study, we examined the internal structure of the Social Desirability Scale using ULSMV method and results support a one factor structure as reported by its authors, *χ^2^* = 1,849.637 (64), CFI = 0.943, uSRMR = 0.045 [90% CI, 0.044; 0.048], RMSEA = 0.155 [0.149; 0.159]. Meanwhile, reliability using omega was *ω = 0*.935(95% CI = 0.929; 0.940). An item example is: “Most people have cheated on an exam, even if it was once in their lives.”

### Procedures

Protocols used in the current study were from three studies approved by the Institutional Review Board of Ponce Health Sciences University (Protocols #150217-ER, #160212-ER, & #160913). Participants in all samples were selected by a convenience non-probabilistic sample method and the inclusion criteria were to be 21 years of age or older and to work at least 20 h per week in different organizations in Puerto Rico. On the other hand, participants were excluded ante-hoc, which included if they did not agree to participate voluntarily.

#### Data analysis

To model GAD-7 items, the researchers used item response theory modeling. Different models for polytomous items were implemented, however the expected performance of the item parameters varied. Discrimination and location (*a* & *b*, respectively) are the parameters, and each model defines them as fixed (restricted) or free. The rating scale (RMS; (48)), partial credit (PCM; (49)), graded response (GRM; (45)), and generalized partial credit (GPCM; (46)) models were used in the study.

The assumption of unidimensionality of the GAD-7 was evaluated with the *automated item selection procedure* (AISP), based on Mokken Scaling Analysis nonparametric IRT (65), in steps of 0.10, from Hi = 0.40 (Hi: coefficients of item scalability; (65)). To verify their results, the *empirical Kaiser criterion* (57) was used, based on the analysis of the inter-item polychoric correlation matrix of the GAD-7. Once the dimensionality of the GAD-7 was defined, the data were fitted to the polytomous models rsm, pcm, grm, and gpcm, and their fit was evaluated at two levels (66): global, and items. First, the global fit was evaluated with the M2* statistic (67), and approximate fit indices, such as RMSEA_2_ (based on M2*; (68)) and SRMR (respectively, with the points cut-off at <0.06, and 0.05; (69)).

The comparative evaluation, that is, the selection of models, was made with the comparison of the Akaike’s Information Criterion (AIC; (70)), Bayesian Information Criterion (BIC; (71)) and Log-Likelihood (LL) indices, but with an emphasis on BIC due to its more frequent accuracy in identifying the correct model (72). These criteria are appropriate for the present study because they show sufficient power since the total sample size and the subsamples were greater than 500 and with more than 5 items (47). In the present study, the total sample size can be considered large (>1,000; (47, 51)), and moderately large in each subsample (>1,000; (47)).

The two best models were further investigated by the distinguishability between them (null hypothesis: the compared models are indistinguishable) and their superiority, or the model with the best fit (Null hypothesis: the chosen model fits better than the compared model), by means of the Vuong test (73). This test is efficient in differentiating models such as GRM and GPCM (74).

The model with satisfactory fit at the global level was chosen to examine the level of fit: of the items, focused on two aspects (75): their adequacy to the model and the violation of local independence through residual covariation between the items (66). The fit of the item to the model was evaluated using generalized S-χ^2^ (76, 77) and RMSEA based on M2*. The criterion was generalized S-χ^2^ *p* value not statistically significant and/or low RMSEA (< 0.06). The assumption of local independence was evaluated with the degree to which residual covariation occurred ((75): (78)). The Jackknife Slope Index (JSI; (78)) was applied to each pair of items, based on the increase in slope parameters when LD exists, and interpreted as a form of Lagrange multiplier test (78). To reduce the Type I error (detection of false covariations due to the influence of the sample size on JSI; (78)), the violation of local independence was detected when the JSI values were greater than their mean plus 2.58 times (corresponding to with *p* < 0.01) the standard deviation of the JSI.

Once the appropriate model was chosen, the efficiency of the slope and location parameters of the items were evaluated (a and b, respectively). Based on Baker and Kim (52), slope strength (a) was identified with cut-off points at: < 0.64 (low), 0.65 to 1.34 (Moderate), 1.35 to 1.69 (High); and > 1.70 (Very high). The location parameters (b1, b2, b3) were evaluated according to Linacre (79), centered on the distance between them (5.0 ≤ *b_i_* ≥, the monoticity of the item-score relationship, and the order of the location parameters (*b1* < *b_2_* < *b_3_*).

As measures of accuracy and/or reliability of the GAD-7 score, graphs of information curves of the items and of the test score were obtained, as well as the marginal and conditional reliability. Differential item functioning (DIF) was examined with a hybrid model (80, 81), where ordinal logistic regression (82) and Theta latent attribute scores are used to examine differences in item response probability. Three models were tested hierarchically: the latent attribute factor (DIF1 model), the effect of group membership (DIF2 model), and the interaction between both predictors (DIF3 model). The two types of DIF (non-uniform and uniform) were tested, respectively, using the statistical difference (Δχ^2^) between DIF3-DIF2, and then DIF2-DIF1. Two criteria were used to identify items with DIF: Δχ^2^ statistically significant at the 0.01 level, and the size of this evaluated difference expressed in pseudo R^2^ coefficients (Cox-Snell-*R*^2^, and Nagelkerke-*R*^2^; (83)) The size of these coefficients was evaluated at three levels (0.02, 0.13 and 0.26: low, moderate, large, respectively; (82)).

The person-fit condition was also evaluated to detect atypical response patterns, not adjusted to the chosen IRT model. This detection is part of a way to identify outlier or inconsistent patterns (84). The Zh index (85) was used, with a cutoff point at Zh < −2.0 for atypical or highly inconsistent responses (84), and Zh > 2.0 for overfitting responses.

In the context of psychosocial health measures (i.e., typical performance), misfitting can occur due to fluctuating answers across domains (e.g., if a participant is experiencing severe issues in one dimension but not in others), distraction, low motivation, and exaggerating good/bad (86–88). Research on the different uses of person fit has highlighted its importance and diagnostic value, and this statistical assessment is quite relevant to screen anxiety with GAD-7.

For the association between the GAD-7 score with external variables, estimates of expected *a posteriori* scores (EAP; (89)) were obtained, which tend to be more recommended to maximize the precision of individual scores (90, 91). To examine the association with external variables in a framework of validity hypotheses and given the application of the results to a population of workers, constructs directly linked to work experience were first chosen. Two of these were work-related rumination (measured by the Work-Related Rumination Scale-Spanish Version; WRRS-SV, (38)), and work engagement (measured by the Utrecht Work Engagement Scale - 3 items, UWES-3; (56)). With the rumination construct, convergent validity was hypothesized, since they are linked to an increase or decrease in anxiety (92, 93). Specifically, a positive and high correlation was hypothesized with affective rumination, and a moderate one with problem-solving pondering and detachment. With engagement, divergent validity was hypothesized, with low and negative correlation, since both come from different types of constructs, that is, one derived from psychosocial factors at work, and one linked to generalized experiences in multiple contexts.

## Results

### Unidimensionality

The AISP nonparametric method indicated that there was no variation in the assignment of the items to more than one homogeneous scale, and therefore the items cannot be partitioned into more than one scale. The scalability coefficients on this single scale can include values of 0.60 or more (Table 2). With the Empirical Kaiser Criterion, the eigenvalues obtained in the sample were 4.9, 0.45, 0.41, 0.39, 0.27, 0.27, 0.21; compared to the random reference generated by the Empirical Kaiser Criterion (1.1, 0.37, 0.35, 0.32, 0.28, 0.27, 0.23), only the former was substantially large (ratio: 10.8) and far removed from the rest of the eigenvalues. Therefore, the latent unidimensionality of GAD-7 is supported.

### Overall fit

The results of the overall fit are presented in Table 3. All models were not statistically significant, indicating that they can be accepted to model GAD-7 responses. However, RSM and PCM showed comparatively the lowest fit indicators, and at this stage they were discarded. The two models with the best fit, based on the information indices (AIC and BIC) and LL, were GRM and GPCM; this was also supported by the approximate CFI (> 0.98), RMSEA (< 0.08), and SRMR (< 0.05) indices. These two models were chosen to be evaluated for differentiability ( $\partial \omega_{*}^{2}$ ) and superiority (z test) between them, with the Vuong test. Both tests were statistically significant, indicating that the GRM model was sufficiently different from the GPCM ( $\partial \omega_{*}^{2}$ , *p* < 0.01) and with a better fit (z, p < 0.01). The GRM model was also compared with a model in which the discrimination parameter of each item (a) was constrained to be equal to each other, approaching the tau-equivalent condition. The $\partial \omega_{*}^{2}$ and z tests were statistically significant (*p* < 0.001), indicating the differentiability between both and the superiority of the GRM fit without equality restrictions.

**Table 3:** Fit of polytomous IRT models for GAD-7

			Fit test and indices					Vuong tests
	M^2^*(df)	RMSEA (90% CI)	SRMR	CFI	AIC	BIC	LL	Distinguishability $\partial \omega_{*}^{2}$	Better fit(*z*)
RSM	466.04 (32)	0.078 (0.071, 0.084)	–	0.981	24831.66	24888.71	−12405.83	–	–
PCM	307.52 (68)	0.080 (0.072, 0.088)	0.05	0.985	24677.03	24802.53	−12316.51	–	–
GPCM	162.07 (14)	0.069 (0.059, 0.078)	0.034	0.992	24519.94	24679.68	−12231.97	0.085**	7.36 **
GRM	155.2877 (14)	0.067 (0.058, 0.077)	0.023	0.993	24317.16	24476.9	−12130.58	–	–
GRM-cons	310.79 (20)	0.080 (0.073, 0.089)	0.049	0.985	24453.98	24579.49	−12204.99	0.085**	5.43**

RSM, rating scale model; PC, partial credit model; GPCM, generalized partial credit model; GRM, graded response model; GRM-cons, GRM with constrained discrimination parameter; AIC, Akaike’s Information Criterion; BIC, Bayesian Information Criterion; LL, Log-likelihood; Vuong test, Distinguishability and better fit of GRM compared with other models (GPCM and GRM-constrained). **p < 0.01.

### Item level fit

Fit to model. Except for item 4 (Table 4), the fit at the item level was statistically significant (p < 0.001); this indicates that the GRM model does not seem to represent the response function of the items. However, the practical fit derived from RMSEA in each item was less than 0.035, indicating that the approximate fit of each item is satisfactory. Therefore, all the items of the GAD-7 were represented by the GR model.

**Table 4:** Parámetros de los ítems, con graded response model (discrimination and location).

Item	Item Fit		Factor Loading	Discrimination and location parameters						
				IRT Parameters					Location Differences (b_i_)	
	S-X^2^	RMSEA_2_	*F*	a se	b_1_ se_1_	b_2_ se_2_	b_3_ se_3_		$\Delta_{1-2}$	$\Delta_{2-3}$
GAD7_1	73.689**	0.024	0.92	3.98	0.13	1.09	1.52		−0.96	−0.43
				0.17	0.02	0.03	0.04			
GAD7_2	104.89**	0.034	0.93	4.40	0.13	1.04	1.59		−0.91	−0.55
				0.20	0.02	0.03	0.04			
GAD7_3	106.91**	0.032	0.90	3.57	−0.13	0.95	1.57		−1.08	−0.62
				0.14	0.03	0.03	0.04			
GAD7_4	38.308	0.009	0.90	3.69	0.02	1.01	1.56		−0.99	−0.55
				0.15	0.02	0.03	0.04			
GAD7_5	95.943**	0.027	0.86	2.89	0.31	1.19	1.78		−0.88	−0.59
				0.12	0.03	0.04	0.05			
GAD7_6	125.70**	0.031	0.82	2.48	0.07	1.15	1.87		−1.08	−0.72
				0.10	0.03	0.04	0.06			
GAD7_7	115.86**	0.030	0.87	3.00	0.49	1.30	1.83		−0.81	−0.53
				0.13	0.03	0.04	0.05			

se, standard error; S-*X*^2^, item-level fit test to graded response model; a, discrimination parameter; b_1_, b_2_, b_3_, location parameter; $\Delta_{i-j}$ , sequential location parameter raw difference; se, standard error; Factor loading, values derivated from IRT discrimination parameter.

**p < 0.01.

### Local independence

The mean JSI was 0.189 and SD = 1.859 and ranged from −2.31 (pair of items 2 and 6) to 4.83 (pair of items 1 and 2); the cut-off point was |4.98|. According to the results presented in Table 5, JSI between items 1 and 2 were close to the cut-off point but it was not higher, so no residual covariation was qualified as problematic. Therefore, it can be concluded here in the absence of local dependency problems.

**Table 5:** Jacknife Slope Index (JSI) for pair ítems of GAD-7.

	GAD7_1	GAD7_2	GAD7_3	GAD7_4	GAD7_5	GAD7_6	GAD7_7
GAD7_1	–						
GAD7_2	4.83	–					
GAD7_3	0.29	2.20	–				
GAD7_4	−1.91	−1.79	−0.10	–			
GAD7_5	−1.13	−2.05	−0.82	3.31	–		
GAD7_6	−1.63	−2.31	1.26	2.10	0.98	–	
GAD7_7	−0.09	−0.00	−0.55	−0.47	0.67	0.1.21	–

### Parameters of items

With the GRM adjusted to the responses to the items, the parameters shown in Table 4 were obtained. The discrimination parameter was >1.71 in all the items, indicating that they were very high. In the factor loading metric, derived from the discrimination parameter a, these were > 0.80 (Min = 0.82, Max = 0.93. M = 0.88), and the proportion of retained variance was 0.791. This strength of the factor loadings can also be considered very high (94). Regarding the location parameter, the thresholds maintained a constant incremental pattern from b1 to b3, indicating the absence of disorder. The progress of the location parameters was, on average, −0.95 and − 0.57, respectively; this increase was below 1.4 (79), but below 5.0, indicating the absence of excessive distancing, but also narrow range of construct coverage.

**Figure 1:**
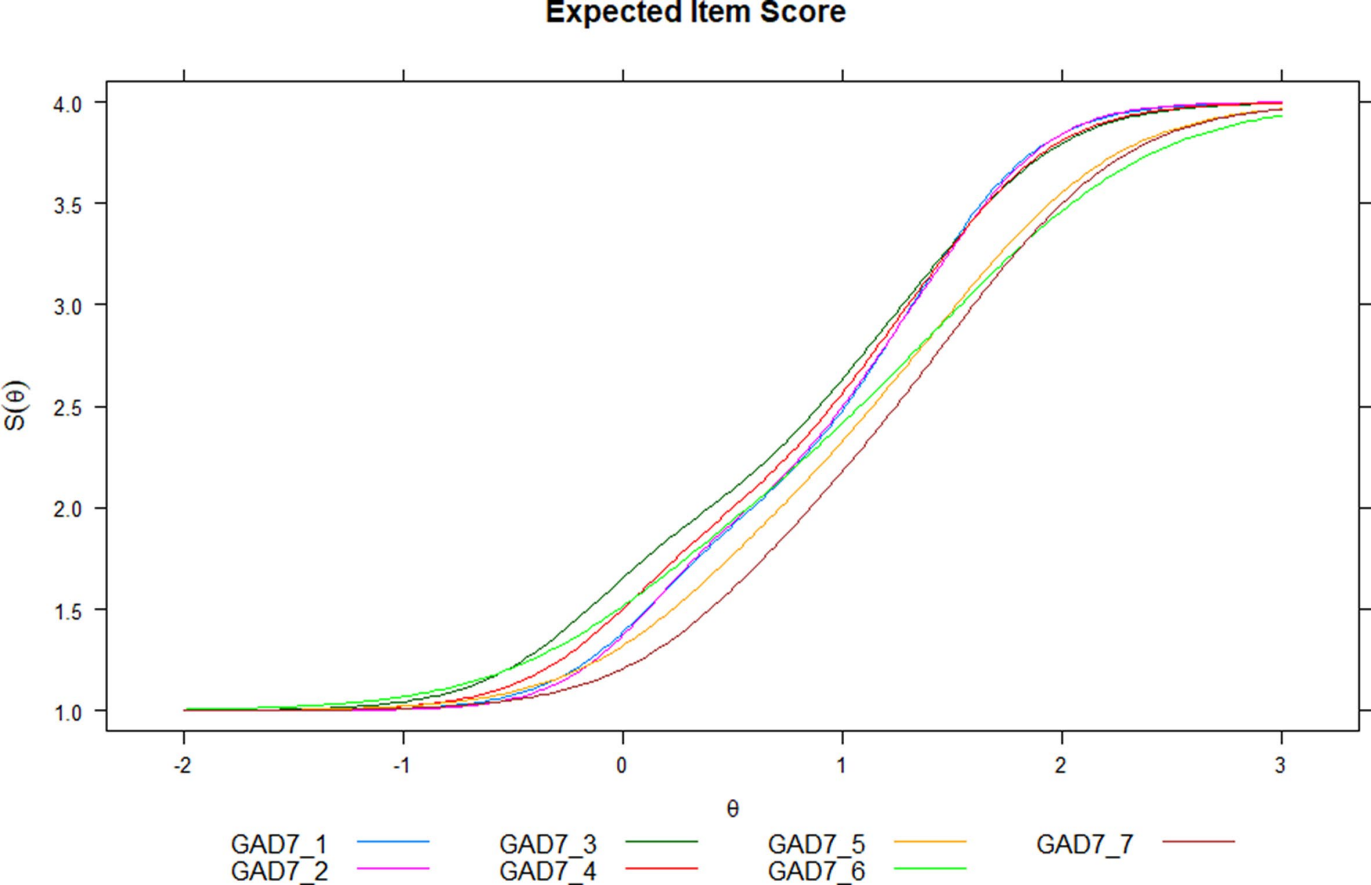
Expected item score.

**Figure 2:**
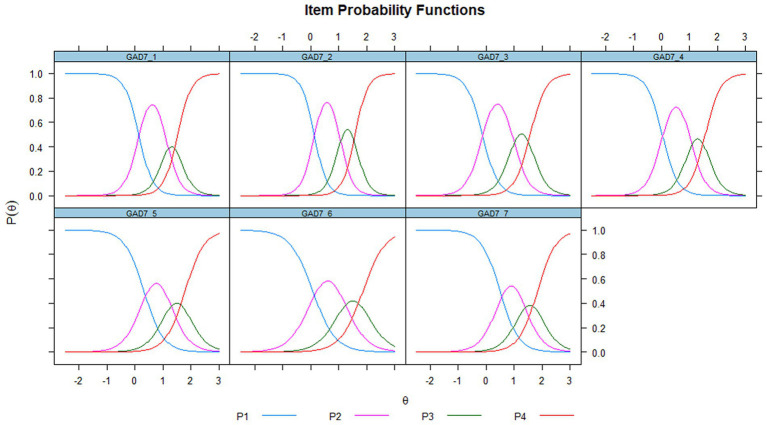
Item characteristic curves (item probability functions). P1, P2, P3, P4: response options “not at all,” “several days,” “more than half the days,” “nearly everyday,” respectively.

On the probability of response of the items along the latent attribute θ, this relationship item-latent variable θ can be seen in Figure 1. An increasing monotonic association is shown between the probability of the response options, along the latent variable θ. Although differences in the curves are observed, they do not appear to be substantially different. In Figure 2, each characteristic option curve shows a clearly differentiated pattern under the GRM model. Approximately, from the mean of the latent attribute, response options equal to or greater than the second option indicate an increase in perceived generalized anxiety.

### Person-fit

A total of 99 participants (4.4%) with Zh scores less than −2 were identified, indicating a response pattern inconsistent with the rest of the 2,219 participants (95.5%). No participant was identified with overfitting responses with Zh > 2.0, suggesting that response similarity, even high similarity, can be seen as a typical response pattern. In line with the evaluation of this individual estimation of person-fit indicators (95), Zh was correlated with external criteria, such as WRRS-SV, UWES-3 scores, and demographic variables (see Table 6). Linear association was essentially zero with each of these variables, suggesting their trivial impact on this association, and that participants may be retained for further analyses.

**Table 6:** Linear association: GAD score and external variables.

Scale	GAD-7 EAP score	Zh score
Work-related rumination scale (WRR)		
*Affective Rumination (AR)*	0.65**	0.04*
	(0.62, 0.67)	(0.00, 0.08)
Problem-solving pondering (PSP)	0.35**	0
	(0.31, 0.39)	(−0.04, 0.04)
*Detachment (Det)*	−0.16**	−0.05**
	(−0.20, 0.12)	(−0.10, −0.01)
*Work engagement (UWES-3)*	−0.17**	−0.02
	(−0.22, −0.12)	(−0.07, 0.02)
Sociodemographic variables		
Sex	0.05*	0.01
	(0.00, 0.09)	(−0.02, 0.05)
Age	−0.15**	0.03
	(−0.19, −0.11)	(−0.00, 0.07)

GAD-7 EAP score, Expected *A Posteriori* scores for GAD-7; Zh score, index for atypical response pattern. *p < 0.05. **p < 0.01. Confidence Intervals in 95%.

### Precision

**Figure 3:**
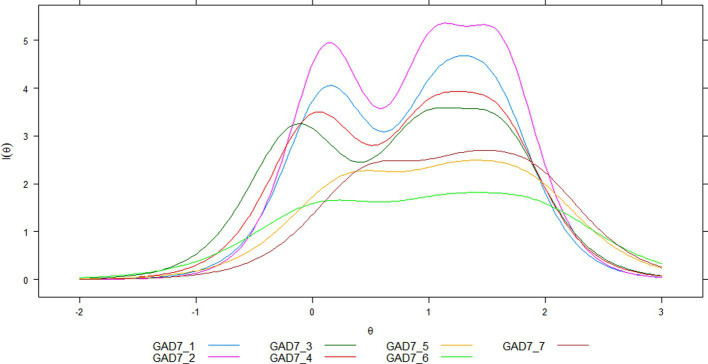
Item information curves for GAD-7.

At the item level, while distinct and monotonically similar option characteristic curves could be recognized, the information curves were different (Figure 3). There are four items (1,2,3 and 4) with two peaks in the mean (*θ* = 0), and around *θ* = 1.0, while the rest of the items (5, 6 and 7), show their curves of highest information between *θ* = 1.0 and 2.0; these items also show the comparatively lower information curves.

**Figure 4:**
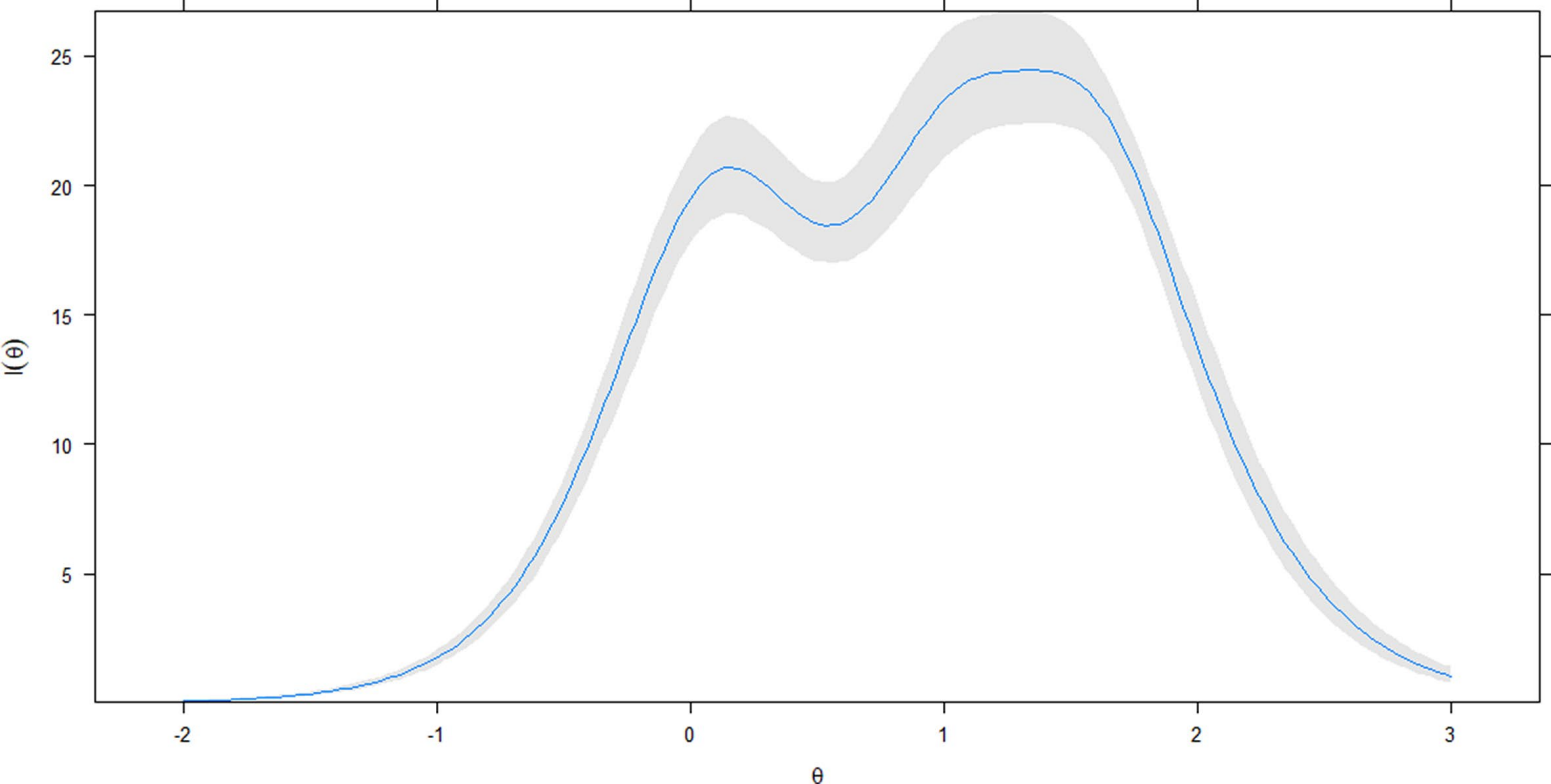
Test information curve of latent construct. Gray area around of test information line: 95% confidence Interval (1,000 bootstrap samples).

**Figure 5:**
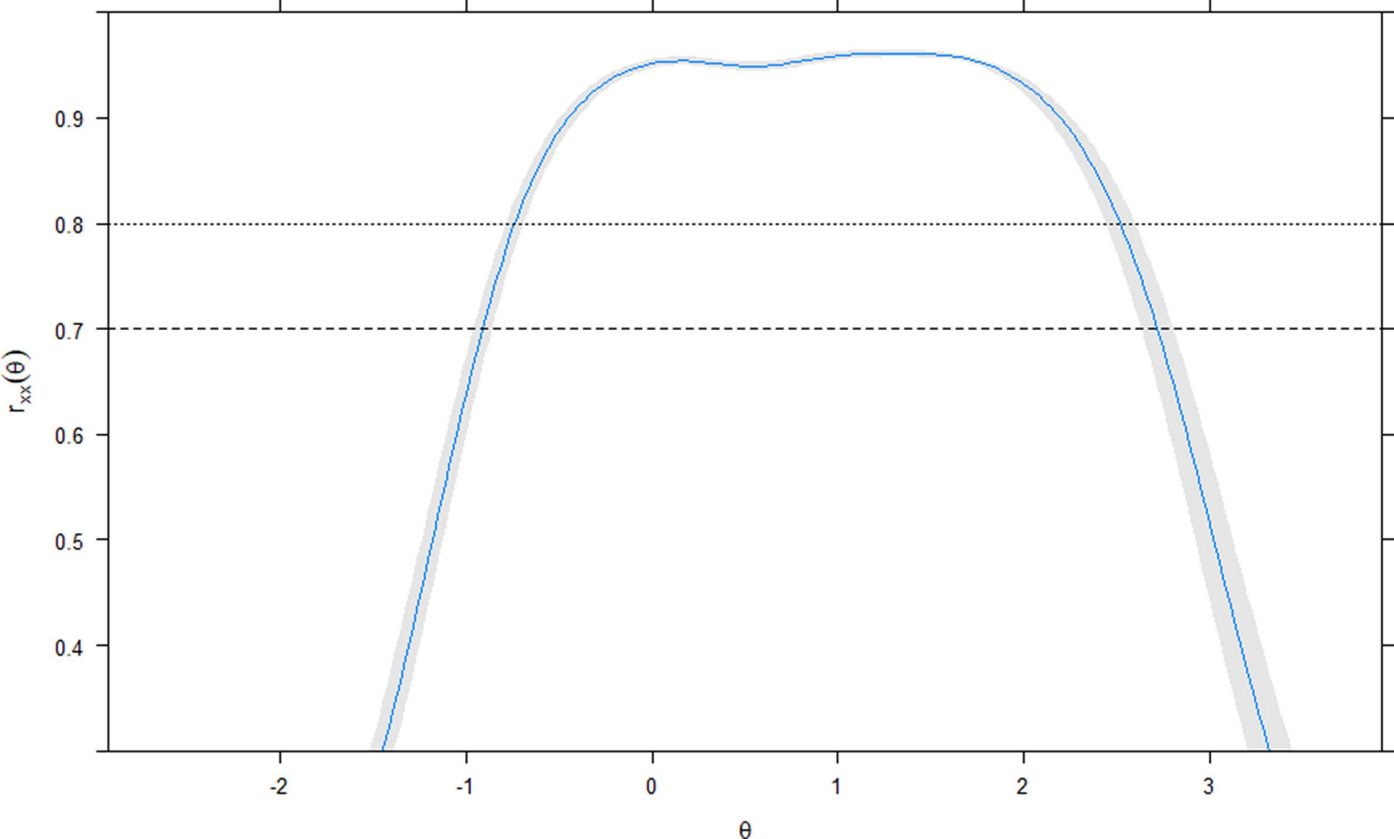
Conditional reliability of GAD-7 latent construct. *r*_xx_(θ): empirical reliability. θ: latent trait.

At the level of the complete scale, the greater informative capacity of the total score globally represented what happened in the items (Figure 4); that is, two peaks of greater precision were observed in the mean (*θ* = 0) and around θ = 1.5. Beyond θ = 2.0, and below θ = 0, the accuracy of the score showed a continuous decrease. In the reliability coefficient metric, the empirical estimate *r_xx_(θ)* = 0.85. Conditional on the levels of the latent attribute θ (Figure 5), values equal to or greater than 0.80 remain in the range close to a θ = −1.0, y θ = 2.5.

### Differential item functioning

Table 7 shows the results of the DIF analysis. The difference tests (Δχ^2^) between the tested ordinal logistic regression models (DIF1, DIF2 and DIF3) indicated that they were not statistically significant (*p* > 0.10) in the evaluation of the non-uniform DIF (DIF3-DIF2 Δχ^2^ < 0.75) and uniform DIF (DIF2-DIF1 Δ*χ*^2^ < 0.85). Along with failing to reject the null hypothesis of no DIF, the effect size indicators (Nagelkerke R^2^ and Cox-Snell *R*^2^) were essentially zero in magnitude. Taken together, these results indicate the absence of DIF with respect to the sex of the participants.

**Table 7:** Differential item functioning (DIF) for GAD-7 (group: sex of participants).

Item	Δχ^2^		Nagelkerke *R*^2^		Cox-Snell *R*^2^	
	DIF_3_-DIF_2_	DIF_2_-DIF_1_	DIF_3_-DIF_2_	DIF_2_-DIF_1_	DIF_3_-DIF_2_	DIF_2_-DIF_1_
GAD7_1	0.06	0.70	0.00	0.00	0.00	0.00
GAD7_2	0.03	0.80	0.00	0.00	0.00	0.00
GAD7_3	0.69	0.00	0.00	0.00	0.00	0.00
GAD7_4	0.02	0.88	0.00	0.00	0.00	0.00
GAD7_5	0.65	0.00	0.00	0.00	0.00	0.00
GAD7_6	0.73	0.19	0.00	0.00	0.00	0.00
GAD7_7	0.25	0.22	0.00	0.00	0.00	0.00

Δ*χ*^2^, difference of *χ*^2^ value of *p* from compared models; DIF1, latent factor model as predictor; DIF2, model with latent factor more group variable as predictors; DIF3, model with latent factor more, group variable, more interaction latent factor *y* group variable as predictors.

### Association with other variables

Table 6 shows the results between the GAD-7 and the chosen variables. The correlation between the Zh of each participant (e.g., indicator of atypical responses) with the external variables is also shown, to identify their impact on this association (see Person-fit section; (95)), their correlations with external variables are also presented.

## Discussion

The objective of the present study was to evaluate the properties of GAD-7 from the IRT perspective, in a sample of workers from Puerto Rico. To avoid inducing its validity from the results of the previous psychometric literature, new IRT-based psychometric parameters were obtained based on the present sample.

The assumptions for applying IRT to the GAD-7 were clearly met with respect to latent unidimensionality and monoticity of item-construct relationships. Through the evaluation of the number of dimensions, the unidimensionality of the GAD-7 was confirmed and is in line with the precedent psychometric findings of this same measure. As in the rest of the GAD-7 validation studies cited, the unidimensionality of the instrument is not altered, and the GAD-7 in the Puerto Rican sample represents the variability of generalized anxiety. According to the EKC, the difference of the first eigenvalue against a possible second factor was extremely large (11.04 times larger), and the strength of the scalability (through the AISP procedure) was maintained until Hi = 0.60, a level considered strong (95). This result is added to the previous studies that also concluded in unidimensionality. Regarding the content of the GAD-7, this indicates that the set of behaviors sampled to represent generalized anxiety in the adult population of workers is explained by a single construct. Apparently, other substantive or method constructs (e.g., (96, 97)) are not necessary to obtain a good representation of anxiety from the items. Therefore, the implication of this finding is that a single dimension is adequate to represent item responses to the GAD-7 and should be the baseline hypothesis when exploring additional dimensions. For example, these dimensions associated with the method. Specifically, the preceding psychometric literature shows that other factorial solutions are possible in the GAD-7: two dimensions (41–43), and unidimensionality with correlated residuals (35). But also, dimensions associated with the item scaling method can occur and be detected by random intercept factor analysis (97).

Regarding the IRT model appropriate to the GAD-7 data, the graded response model (GRM) was the most appropriate to obtain the parameters of the items. Other models that closely competed with the fit were GPCM and GRM with the specification of equal factor loadings. As noted in the review and analysis of the literature, due to the absence of a wide range of models oriented to ordinal items, this analysis was done. This implies that the GAD-7 items show different intensities in the location and discrimination parameters, and therefore cannot be treated as statistically equivalent. The equality constraint on factor loadings, which represents a tau-equivalent model (98), confirmed this conclusion regarding the unequal validity of the items with their construct. Although the parameters of the items can be seen as very similar, through the Vuong (73) tests, the GRM model achieved a better fit and sufficient distinguishability. Two implications follow from this: that the GRM can help effectively build a working hypothesis for studies where different IRT models are tested for ordinal items, and that a more accurate estimate of score reliability requires a coefficient other than alpha, because to its well-known assumption of tau-equivalence in its items (98).

In the local independence inspection, according to the established criteria (−4.98 < JSI > 4.98), no violation of this problem was found. But the residual covariation between items 1 and 2 was close to the criterion. Since the detection of local dependence can change according to the criterion that the researcher chooses, that is, a liberal one or a conservative one, it would be advisable to pay attention to this specific covariation. However, both items are also included in an abbreviated version of the GAD, the GAD-2 (99), whose content is accepted as the core symptoms of generalized anxiety and is accepted as a good screening measure. (44). Both items seem to share additional variance to the latent construct and may be significant indicators of anxiety as a similar but different unit to the rest of the GAD-7 items. This does not suggest the transferability of the validity results of the GAD-2 from the GAD-7 in the present study, and it is necessary to avoid inferring its validity from our results with the GAD-7 in workers (36, 37).

The adjustment of each item to the GRM was satisfactory, with high factor loads (between 0.82 and 0.93); this high amount of validity of each item with its construct was equivalent to the discrimination parameter (a), which were also high. Regarding the response thresholds, their location in the latent attribute was similar, and the amount of difference between them also tended to be similar. Together with the monoticity of their ordering, as a whole, the GAD-7 items are highly valid and apparently function similarly. This pattern of similarity was also observed in the DIF analysis, where all items showed a trivial amount of DIF.

An apparent distinction between the items was the information function, in which items 1 and 2 showed some information, and closely so did item 4. In general, the items seem to retain more information at approximately level 0 of the latent attribute (i.e., about the mean), and 1.5SD above the mean. Both levels of latent attribute are optimal for detecting workers with average and high generalized anxiety. Due to the high similarity of the discrimination and location parameters between the GAD-7 items, the similarity in the item information curves also occurred in a similar test information curve pattern. The GAD-7 latent score shows its best accuracy between the mean and 1.5SD, with two peaks at these latent attribute points. In the reliability metric, the reliability range at these points varies between approximately 0.90 and 0.95, values that usually favor the clinical use of a measure (100).

As part of the evaluation of the IRT model on the data, the detection of atypical or aberrant responses resulted in a small percentage of participants (< 5.0%). This low percentage may suggest that the interaction between the conditions of the application of the instruments, and personal disposition, did not have a substantial impact on the expected responses to the GAD-7. This may imply that the measurement of generalized anxiety, with adequate measurement conditions, adds clinical value to the effects of psychosocial factors at work. The effective impact of these outlier scores on the investigation of the association with the external variables was innocuous, and it was not necessary to remove these participants. However, the value of this detection is important in moving to a next step to determine the cause and effective treatment of this problem on an individualized basis (84, 85). It is also relevant to indicate that no participant was detected as having overfitting responses (Zh > 2), suggesting that the high response similarity is a typical response pattern with respect to GAD-7. This is reasonable given the similarity of the item parameters.

In terms of the relationship between GAD-7 and other measures, the GAD-7 correlated negatively as expected with work engagement and the detachment subscale of the WRRS-SP. There are some studies (e.g., (64)) that have found also a negative and small relationship between anxiety and work engagement, which argue that work engagement is seen as an antithesis to the more familiar and investigated term, ‘burnout.’ Meanwhile, the relationship between the scores GAD-7 and the detachment subscale of the WRRS-SV was also small and negative, which is like the results found by Rosario-Hernández et al. (38). On the other hand, the relationship between the scores of the GAD-7 and the other two subscales of the WRRS-SV, affective rumination, and problem-solving pondering, were positives and considered as large and medium in size, respectively. These results are very similar in size to those obtained by Rosario-Hernández et al. (38) who found significant correlations between anxiety with affective rumination (r = 0.704, *p* < 0.001) and problem-solving pondering (r = 0.378, p < 0.001), respectively. Moreover, it can be noticed the emotional arousal contrast in the correlation size of affective rumination when compared to the correlation size of problem-solving pondering with anxiety (101). We can use the conservation of resources (COR; (62)) to help explain these relationships. Thus, the primary concept of the COR theory is that people have a strong desire to acquire, hold onto, and preserve the resources they value (62). The novel component of COR theory is that it not only describes what people do under stress, but also how they act in the absence of threats. In particular, the model predicts that people will want to reduce the net loss of resources when under stress. In contrast, people work to create resource surpluses when there are no risks to offset the possibility of future loss. Work engagement could be seen as a resource surplus, in contrast to the COR theory’s description of burnout as a condition of extreme resource depletion (102, 103). When people have resource surpluses, they are more likely to have good health and well-being (64).

To put in context these relationships, thoughts about work-related concerns that are recurrent in nature are referred to as work-related rumination (101). Rumination can have serious negative effects on one’s health and well-being, for example, there are some studies (e.g., (12, 38, 104)) that have found a relationship between affective rumination and anxiety. In addition, Rosario-Hernández et al. (38) found that the relationship between anxiety and the three components of work-related rumination (affective rumination, problem-solving pondering, and detachment: AR, PSP, and Det, respectively) proposed by Cropley & Zijlstra (101) obtained 0.704, 0.378, and − 0.150, respectively. Moreover, results from our study, in terms of correlation coefficient sizes are very similar to those obtained by Rosario-Hernández et al. (38) and it is particularly interesting to point out that the correlation between anxiety and affective rumination are higher than those of anxiety and problem-solving pondering, which can be attributed to emotional component that affective rumination characterizes it and differentiates it from PSP.

What are the practical implications when workers are detected with high anxiety in their work organizations? According to Greden et al. (105), stress and anxiety are statistically the most frequently reported mental health issues at work. They continue by saying that these workers are unproductive, prone to accidents, plagued by errors, use of sick leave, and turnovers. Workplaces are increasingly being targeted in attempts to promote mental health promotion, prevention, and interventions due to the high prevalence of mental health issues among working individuals (106). Despite the fact that employee assistance programs (EAPs) have become increasingly common over the past few decades and that the majority of mental health conditions have effective evidence-based treatments that are well-known and frequently accessible, EAPs typically do not offer mental illness prevention or support for employees who are already experiencing mental illness. This indicates that many people do not get the care they require (106). Linked with this, Carrol found that employees were hesitant to utilize accessible counseling services if they felt it might affect their career opportunities. As a result, workers run the danger of delaying seeking treatment until their symptoms are more severe and leading to clinically substantial functional impairments.

However, by early identification, connection to treatment, greater worker productivity, and lower health insurance costs, measuring employee psychological discomfort and screening them for mental diseases has a significant potential to benefit both the employees and the organization. Moreover, Wang et al. found that systematic assessment to identify and treat effectively mental illness significantly improves not only clinical outcomes but also workplace outcomes. For example, the Japanese government launched a mandatory occupational health policy called the Stress Check Program, which mandates that all workplaces with 50 or more employees run the program at least once per year. Thus, the program requires an employer to perform a psychosocial stress screening for workers; provide individual employees with their results; arrange an-interview with a health professional for high-stress workers who want one; and make efforts to improve the overall work environment based on a group analysis. In this sense, Taubman et al. (106) indicate that workforce screening and assessment is therefore practical and provides useful data on the employee population. Employees are less likely to feel stigmatized for participating in screening or intervention programs that are available to all employees at their organization. These screening and intervention have the capacity to simultaneously reduce workplace-based risk factors for mental health, capture everyone who may develop a mental illness in the future as well as those who are currently unidentified or untreated and reduce workplace-based risk factors for mental health. The health system has discovered that such screening can reduce costs, which is unexpected given that this is frequently the top priority for enterprises.

The results obtained must be seen within the framework of the limitations of the study. One limitation is the representativeness of the sample of workers, with respect to the population of workers in Puerto Rico. Although the results can be considered stable compared to other studies, the variation between defined groups of workers, in a multilevel setting, was not directly explored. In this sense, the intensity with which workers are exposed to negative psychosocial factors at work is linked to their effects on negative emotionality (such as anxiety). However, this intensity of the anxiety experience within-groups of workers cannot be assumed to be the same between-groups of workers. This has the consequence that, in a measurement model, even when the item-dimension relationship is the same between groups (metric or weak invariance), the different intensity in the construct can influence different intercepts, which would violate the invariance of this parameter. [scalar or strong invariance]. Multilevel modeling may be a new methodological avenue to explore this (107). Another limitation was that a method factor was not included, for example, the random intercept factor (96, 97), a model that represents the variability in the use of the scale of response and that can capture response patterns independent of the content of the GAD-7. However, given the strength of the fit indicators, and the person-fit assessment, their possible presence could have a trivial impact on the interpretation of the conclusions. Another limitation was that we did not include a psychosocial work factor to link it with the anxiety measured by the GAD-7. Our study did include a construct associated with work experience and its links with anxiety, such as the WRR. In the context of occupational health, WRR is an individual-variability factor that interacts with psychosocial factors external to the individual.

## Conclusion

The GAD-7 items are represented by a unidimensional construct, and with the graded response model as the most appropriate item response theory measurement model. Each item also shows a satisfactory fit to this model. The items show high similarity in the item-construct relationship, and moderately strong similarity in the scaling of the response options. No violation of local independence was detected, and the person-fit analysis observed less than 4% of participants with atypical responses; these atypical responses no longer had a trivial impact on the evaluation of the relationship with other variables. The precision of the scores occurs around the mean and one standard deviation above the mean, something that is also repeated in the items. An absence of item differential functioning was obtained in the grouping by sex. Finally, the association with the constructs of rumination, work engagement, and sociodemographic variables (sex and age) supported convergence and divergence validity. The GAD-7 is an optimal tool for screening the intensity of anxiety symptoms in Puerto Rican workers.

## Data availability statement

The original contributions presented in the study are included in the article/supplementary material, further inquiries can be directed to the corresponding authors.

## Ethics statement

The studies involving human participants were reviewed and approved by Ponce Health Sciences University. The patients/participants provided their written informed consent to participate in this study.

## Author contributions

CM-S, ER-H, LR-M, and MA-R: conceptualization, methodology, writing-original draft preparation, and writing-review and editing. CM-S and ER-H: formal analysis and supervision. ER-H and LR-M: investigation. ER-H: funding acquisition. All authors contributed to the article and approved the submitted version.

## Funding

“The project described was supported by the RCMI Program Award Number U54MD007579 from the National Institute on Minority Health and Health Disparities. The content is solely the responsibility of the authors and does not necessarily represent the official views of the National Institutes of Health.”

## Conflict of interest

The authors declare that the research was conducted in the absence of any commercial or financial relationships that could be construed as a potential conflict of interest.

## Publisher’s note

All claims expressed in this article are solely those of the authors and do not necessarily represent those of their affiliated organizations, or those of the publisher, the editors and the reviewers. Any product that may be evaluated in this article, or claim that may be made by its manufacturer, is not guaranteed or endorsed by the publisher.

## References

[1] Canino G Shrout PE NeMoyer A Vila D Santiago KM García P. A comparison of the prevalence of psychiatric disorders in Puerto Rico with the United States and the Puerto Rican population of the United States. Soc Psychiatry Psychiatr Epidemiol. (2019) 54:369–8. doi: 10.1007/s00127-019-01653-6, PMID: 30649577 PMC6440857

[2] Puerto Rico Departemnt of Health. (2021). Estadística de Pacientes Revisado 31 de Agosto de 2021 [patients statistics] available at: https://www.salud.gov.pr/CMS/DOWNLOAD/5458 (accessed August 31, 2021)

[3] Rodríguez-Kierce J. Healthcare in Puerto Rico: challenges and our big opportunity V2A consulting Insight, (2023). available at: https://v2aconsulting.com/wp-content/uploads/2023/01/HealthcareinPR_v2AInsightspar5_pdf-5.pdf (accessed February 25, 2023)

[4] Wittchen HU Kessler RC Beesdo K Krause P Hofler M Hoyer J. Generalized anxiety and depression in primary care: prevalence, recognition, and management. J Clin Psychiatry. (2002) 63:24–34. 12044105

[5] Spielberger CD Reheiser EC Owen AE Sydeman SJ. Measuring the psychological vital signs of anxiety, anger, depression, and curiosity in treatment planning and outcomes assessment In: Maruish ME, editor. The use of psychological testing for treatment planning and outcomes assessment: Instruments for adults. Mahwah: Lawrence Erlbaum Associates Publishers (2004)

[6] Linden M Muschalla B. Anxiety disorders and workplace-related anxieties. J Anxiety Disord. (2007) 21:467–4. doi: 10.1016/j.janxdis.2006.06.006, PMID: 16890399

[7] Peters E Spanier K Radoschewski FM Bethge M. Influence of social support among employees on mental health and work ability: a prospective cohort study in 2013-15. Eur J Pub Health. (2018) 28:819–3. doi: 10.1093/eurpub/cky067, PMID: 29668870

[8] Deguchi Y Iwasaki S Niki A Kadowaki A Hirota T Shirahama Y. Relationships between occupational stress, change in work environment during the COVID-19 pandemic, and depressive and anxiety symptoms among non-HealthcareWorkers in Japan: a cross-sectional study. Int J Environ Res Public Health. (2022) 19:983. doi: 10.3390/ijerph19020983, PMID: 35055803 PMC8775764

[9] Ortiz VG Juárez-García A. Working conditions and effort-reward imbalance in Latin America In: Siegrist J Wahrendorf M, editors. Work stress and health in a globalized economy: Aligning perspectives on health, safety and well-being. Cham: Springer (2016)

[10] Zinchuk M Kustov G Pashnin E Gersamia A Rider F Yakovlev A. Validation of the generalized anxiety Disorder-7 (GAD-7) in Russian people with epilepsy. Epilep Behav. (2021) 123:108269. doi: 10.1016/j.yebeh.2021.108269, PMID: 34500434

[11] Kim SW Ha J Lee JH Yoon JH. Association between job-related factors and work-related anxiety, and moderating effect of decision-making Authority in Korean Wageworkers: a cross-sectional study. Int J Environ Res Public Health. (2021) 18:5755. doi: 10.3390/ijerph18115755, PMID: 34071991 PMC8197820

[12] Rosario-Hernández E Rovira-Millán LV Sánchez-García NC Padovani Rivera CM Velázquez Lugo A Maldonado Fonseca IM. A boring story about work: do bored employees ruminate? Revista Puertorriqueña de Psicología. (2020) 31:92–8.

[13] Rosario-Hernández E Rovira-Millán LV Pagán-Torres OM Feliciano Toro BP Cepeda Fax S Martínez N. The effects of workaholism on psychological well-being and the mediating effects of rumination. Conference presented at the 14th Caribbean congress of psychology, Universidad Autónoma de Santo Domingo and Universidad O&M, Santo Domingo, Dominican Republic (2017).

[14] Rosario-Hernández E Rovira-Millán LV Rodríguez Irizarry A Rivera Alicea BE Fernández López LN López Miranda RI. La salud cardiovascular y su relación con los factores de riesgo psicosociales en una muestra de personas empleadas en Puerto Rico. Revista Puertorriqueña de Psicología. (2014) 25:98–116.

[15] Beck AT Steer RA. Beck anxiety inventory manual. San Antonio, TX: The Psychological Corporation Harcourt Brace and Company (1993).

[16] Goldberg DP. Manual of the general health questionnaire. Windsor: NFER Publishing Company (1978).

[17] Spielberger CD Gorsuch RL Lushene RE. STAI manual for the state-trait-anxiety inventory (Self-evaluation questionnaire). Palo Alto: Consulting Psychologists Press (1970).

[18] Erickson SR Guthrie S VanEtten-Lee M Himle J Hoffman J Santos SF. Severity of anxiety and work-related outcomes of patients with anxiety disorders. Depress Anxiety. (2009) 26:1165–71. doi: 10.1002/da.20624 19842165

[19] Spitzer RL Kroenke K Williams JBW Löwe B. A brief measure for assessing generalized anxiety disorder: the GAD-7. Arch Intern Med. (2006) 166:1092–7. doi: 10.1001/archinte.166.10.1092 16717171

[20] Sapra A Bhandari P Sharma S Chanpura T Lopp L. Using generalized anxiety Disorder-2 (GAD-2) and GAD-7 in a primary care setting. Cureus. (2020) 12:e8224. doi: 10.7759/cureus.8224, PMID: 32582485 PMC7306644

[21] Mughal AY Devadas J Ardman E Levis B Go VF Gaynes BN. A systematic review of validated screening tools for anxiety disorders and PTSD in low to middle income countries. BMC Psychiatry. (2020) 20:338. doi: 10.1186/s12888-020-02753-3, PMID: 32605551 PMC7325104

[22] Simpson W Glazer M Michalski N Steiner M Frey BN. Comparative efficacy of the generalized anxiety disorder 7-item scale and the Edinburgh postnatal depression scale as screening tools for generalized anxiety disorder in pregnancy and the postpartum period. Can J Psychiatry. (2014) 59:434–16. doi: 10.1177/070674371405900806, PMID: 25161068 PMC4143300

[23] American Educational Research Association [AERA], the American Psychological Association [APA], & National Council on Measurement in Education [NCME]. Standards for educational and psychological testing: National Council on measurement in education. Washington DC: American Educational Research Association (2014).

[24] Ahn J-K Kim Y y Choi KH. The psychometric properties and clinical utility of the Korean version of GAD-7 and GAD-2. Front Psychol. (2019) 10:127. doi: 10.3389/fpsyt.2019.00127, PMID: 30936840 PMC6431620

[25] Camargo L Herrera-Pino J Shelach S Soto-Añari M Porto MF Alonso M. GAD-7 generalised anxiety disorder scale in Colombian medical professionals during the COVID-19 pandemic: construct validity and reliability. Revista Colombiana de Psiquiatría (English ed). (2021a). doi: 10.1016/j.rcp.2021.06.003, PMID: [Epub ahead of print]. 37863769

[26] Franco-Jimenez RA Nuñez-Magallanes A. Propiedades psicométricas del GAD-7, GAD-2 y GAD-Mini en universitarios peruanos. Propósitos y Representaciones. (2022) 10:e1437. doi: 10.20511/pyr2022.v10n1.1437

[27] García-Campayo J Zamorano E Ruiz MA Pardo A Pérez-Páramo M López- Gómez V. Cultural adaptation into Spanish of the generalized anxiety Disorder-7 (GAD-7) scale as a screening tool. Health Qual Life Outcom. (2010) 8:8. doi: 10.1186/1477-7525-8-8, PMID: 20089179 PMC2831043

[28] Jordan P Shedden-Mora MC Löwe B. Psychometric analysis of the generalized anxiety disorder scale (GAD-7) in primary care using modern item response theory. PLoS One. (2017) 12:e0182162. doi: 10.1371/journal.pone.0182162, PMID: 28771530 PMC5542568

[29] Mills SD Fox RS Malcarne VL Roesch SC Champagne BR y Sadler GR. The psychometric properties of the generalized anxiety Disorder-7 scale in Hispanic Americans with English or Spanish language preference. Cult Divers Ethn Minor Psychol. (2014) 20:463–8. doi: 10.1037/a0036523, PMID: 25045957 PMC4129392

[30] Moreno AL DeSousa DA Souza AM Manfro GG Salum GA Koller SH. Factor structure, reliability, and item parameters of the Brazilian-Portuguese version of the GAD-7 questionnaire. Temas Em Psicología. (2016) 24:367–6. doi: 10.9788/TP2016.1-25

[31] Ruiz MA Zamorano E García-Campayo J Pardo A Freire O y Rejas J. Validity of the GAD-7 scale as an outcome measure of disability in patients with generalized anxiety disorders in primary care. J Affect Disord. (2011) 128:277–6. doi: 10.1016/j.jad.2010.07.010, PMID: 20692043

[32] Seo J-G Park S-P. Validation of the generalized anxiety Disorder-7 (GAD-7) and GAD-2 in patients with migraine. J Headache Pain. (2015) 16:97–7. doi: 10.1186/s10194-015-0583-8, PMID: 26596588 PMC4656257

[33] Zhang C Wang T Zeng P Zhao M Zhang G Zhai S. Reliability, validity, and measurement invariance of the general anxiety disorder scale among Chinese medical university students. Front Psychol. (2021) 12:648755. doi: 10.3389/fpsyt.2021.648755/full, PMID: 34093269 PMC8170102

[34] Zhong Q-Y Gelaye B Zaslavsky AM Fann JR Rondon MB Sánchez SE. Diagnostic validity of the generalized anxiety disorder - 7 (GAD-7) among pregnant women. PLoS One. (2015) 10:e0125096–17. doi: 10.1371/journal.pone.0125096, PMID: 25915929 PMC4411061

[35] Bártolo A Monteiro S Pereira A. Factor structure and construct validity of the generalized anxiety disorder 7-item (GAD-7) among Portuguese college students. Cad Saude Publica. (2017) 33:e00212716. doi: 10.1590/0102-311X00212716, PMID: 28977285

[36] Merino-Soto C Angulo-Ramos M. Metric studies of the compliance questionnaire on rheumatology (CQR): a case of validity induction? Reumatología Clínica. (2021a). doi: 10.1016/j.reuma.2021.03.004, PMID: 34531170

[37] Merino-Soto C Angulo-Ramos M. Validity induction: comments on the study of compliance questionnaire for rheumatology. Revista Colombiana de Reumatología. (2021b) 28:312–3. doi: 10.1016/j.rcreue.2020.05.013

[38] Rosario-Hernández E Rovira-Millán LV Merino-Soto C. Review of the internal structure, psychometric properties, and measurement invariance of the work-related rumination scale – Spanish version. Front Psychol. (2021) 12:774472. doi: 10.3389/fpsyg.2021.774472, PMID: 34899526 PMC8656259

[39] Pagán-Torres OM González-Rivera JA Rosario-Hernández E. Reviewing the psychometric properties and factor structure of the generalized anxiety Disorder-7 (GAD-7) in a sample of Puerto Rican adults. Int J Rec Sci Res. (2020) 11:36885–8. doi: 10.24327/ijrsr.2020.1101.5017

[40] Monterrosa-Blanco A Cassiani-Miranda CA Scoppetta O Monterrosa-Castro A. Generalized anxiety disorder scale (GAD-7) has adequate psychometric properties in Colombian general practitioners during COVID-19 pandemic. Gen Hosp Psychiatry. (2021) 70:147–8. doi: 10.1016/j.genhosppsych.2021.03.013, PMID: 33840481 PMC8807139

[41] Beard C Björgvinsson T. Beyond generalized anxiety disorder: psychometric properties of the GAD-7 in a heterogeneous psychiatric sample. J Anxiety Disord. (2014) 28:547–2. doi: 10.1016/j.janxdis.2014.06.002, PMID: 24983795

[42] Portman ME Starcevic V Beck AT. Challenges in assessment and diagnosis of generalized anxiety disorder. Psychiatr Ann. (2011) 41:79–85. doi: 10.3928/00485713-20110203-06

[43] Kertz SJ Bigda-Peyton J Björgvinsson T. Validity of the GAD-7 in an acute psychiatric setting. Clin Psychol Psychother. (2013) 20:456–4. doi: 10.1002/cpp.1802, PMID: 22593009

[44] Plummer F Manea L Trepel D McMillan D. Screening for anxiety disorders with the GAD-7 and GAD-2: a systematic review and diagnostic metaanalysis. Gen Hosp Psychiatry. (2016) 39:24–31. doi: 10.1016/j.genhosppsych.2015.11.005, PMID: 26719105

[45] Samejima F. Estimation of latent ability using a response pattern of graded scores. Psychometrika Monograph Supplement. (1969) 34:1–97. doi: 10.1007/BF03372160

[46] Muraki E. A generalized partial credit model: application of an EM algorithm. Appl Psychol Meas. (1992) 16:159–6. doi: 10.1177/014662169201600206

[47] Dai S Vo TT Kehinde OJ He H Xue Y Demir C. Performance of Polytomous IRT models with rating scale data: an investigation over sample size, instrument length, and missing data. Front Educ. (2021) 6:721963. doi: 10.3389/feduc.2021.721963

[48] Andrich D. Application of a psychometric rating model to ordered categories which are scored with successive integers. Appl Psychol Meas. (1978) 2:581–4. doi: 10.1177/014662167800200413

[49] Masters GN. A Rasch model for partial credit scoring. Psychometrika. (1982) 47:149–4. doi: 10.1007/BF02296272

[50] Nguyen TH Han HR Kim MT Chan KS. An introduction to item response theory for patient-reported outcome measurement. The Patient. (2014) 7:23–35. doi: 10.1007/s40271-013-0041-0, PMID: 24403095 PMC4520411

[51] De Ayala RJ. The theory and practice of item response theory. New York, NY: Guilford Publications (2013).

[52] Baker FB Kim S-H. The basics of item response theory using R. Pennsylvania: Springer International Publishing (2017).

[53] Hinz A Klein AM Brähler E Glaesmer H Luck T Riedel-Heller SG. Psychometric evaluation of the generalized anxiety disorder screener GAD-7, based on a large German general population sample. J Affect Disord. (2017) 210:338–4. doi: 10.1016/j.jad.2016.12.012, PMID: 28088111

[54] Gong Y Zhou H Zhang Y Zhu X Wang X Shen B. Validation of the 7-item generalized anxiety disorder scale (GAD-7) as a screening tool for anxiety among pregnant Chinese women. J Affect Disord. (2021) 282:98–103. doi: 10.1016/j.jad.2020.12.129, PMID: 33401129

[55] Rosario-Hernández E Rovira-Millán LV Vega Vélez S Zeno-Santi R Farinacci García P Centeno Quintana L. Exposure to workplace bullying and suicidal ideation: an exploratory study. J Appl Struct Equat Model. (2019) 3:55–75. doi: 10.47263/JASEM.3(1)06

[56] Schaufeli WB Shimazu A Hakanen J Salanova M De Witte H. An ultra-short measure for work engagement: the UWES-3 validation across five countries. Eur J Psychol Assess. (2019) 35:577–1. doi: 10.1027/1015-5759/a000430

[57] Braeken J van Assen MAL. An empirical Kaiser criterion. Psychol Methods. (2017) 22:450–6. doi: 10.1037/met0000074, PMID: 27031883

[58] Schaufeli WB Salanova M González-Romá V Bakker AB. The measurement of engagement and burnout: a two sample confirmatory factor analytic approach. J Happ Stud. (2002) 3:71–92. doi: 10.1023/A:1015630930326

[59] A C-D l C Merino-Soto C Reyes-Ayala IF Luna-Yon KV. Validación en trabajadores peruanos de la versión ultra reducida del Utrech Work Engagement. Archivos de Prevención de Riesgos Laborales. (2022) 25:25–33. doi: 10.12961/aprl.2022.25.01.03, PMID: 35037748

[60] Merino-Soto C Juárez-García A Salinas-Escudero G Toledano-Toledano F. Item-level psychometric analysis of the psychosocial processes at work scale (PROPSIT) in workers. Int J Environ Res Public Health. (2022) 19:7972. doi: 10.3390/ijerph19137972, PMID: 35805629 PMC9265707

[61] Cropley M Michalianou G Pravettoni G Millward LJ. The relation of post-work ruminative thinking with eating behaviour. Stress Health. (2012) 28:23–30. doi: 10.1002/smi.1397, PMID: 22259155

[62] Hobfoll SE. The influence of culture, community, and the nested-self in the stress process: advancing conservation of resources. J Appl Psychol. (1989) 50:337–1. doi: 10.1111/1464-0597.00062

[63] Rosario-Hernández E Rovira-Millán LV. Desarrollo y validación de una escala para medir las actitudes hacia el retiro. Revista Puertorriqueña de Psicología. (2002) 13:45–60.

[64] Innstrand ST Langballe EM Falkum E. A longitudinal study of the relationship between work engagement and symptoms of anxiety and depression. Stress Health. (2012) 28:1–10. doi: 10.1002/smi.1395, PMID: 22259153

[65] Mokken RJ. A theory and procedure of scale analysis with applications in political research. Berlin, New York: De Gruyter Mouton (1971).

[66] Reise SP. Item response theory In: Cautlin RL Lilenfeld SO, editors. The encyclopedia of clinical psychology. New York: John Wiley & Sons, Inc. (2015)

[67] Cai L Hansen M. Limited-information goodness-of-fit testing of hierarchical item factor models. Br J Math Stat Psychol. (2013) 66:245–6. doi: 10.1111/j.2044-8317.2012.02050.x, PMID: 22642552 PMC3760206

[68] Maydeu-Olivares A Joe H. Limited and full information estimation and goodness-of-fit testing in 2^n^ contingency tables: a unified framework. J Am Stat Assoc. (2005) 100:1009–20. doi: 10.1198/016214504000002069

[69] Maydeu-Olivares A Joe H. Assessing approximate fit in categorical data analysis. Multivar Behav Res. (2014) 49:305–8. doi: 10.1080/00273171.2014.911075 26765800

[70] Akaike H. A new look at the statistical model identification. IEEE Trans Autom Control. (1974) 19:716–3. doi: 10.1109/TAC.1974.1100705

[71] Schwarz G. Estimating the dimension of a model. Ann Stat. (1978) 6:461–4. doi: 10.1214/aos/1176344136

[72] Kang T Cohen AS Sung H-J. Model selection indices for Polytomous items. Appl Psychol Meas. (2009) 33:499–8. doi: 10.1177/0146621608327800

[73] Vuong QH. Likelihood ratio tests for model selection and non-nested hypotheses. Econometrica. (1989) 57:307–3. doi: 10.2307/1912557

[74] Schneider L Chalmers RP Debelak R Merkle EC. Model selection of nested and non-nested item response models using Vuong tests. Multivar Behav Res. (2020) 55:664–4. doi: 10.1080/00273171.2019.1664280, PMID: 31530187

[75] Chen W-H Thissen D. Local dependence indexes for item pairs using item response theory. J Educ Behav Stat. (1997) 22:265–9. doi: 10.2307/1165285

[76] Kang T Chen TT. Performance of the generalized S-X^2^ item fit index for Polytomous IRT models. J Educ Meas. (2008) 45:391–406. doi: 10.1111/j.1745-3984.2008.00071.x

[77] Kang T Chen TT. Performance of the generalized S-X^2^ item fit index for the graded response model. Asia Pacific Educ Rev. (2011) 12:89–96. doi: 10.1007/s12564-010-9082-4

[78] Edwards MC Houts CR Cai L. A diagnostic procedure to detect departures from local independence in item response theory models. Psychol Methods. (2018) 23:138–9. doi: 10.1037/met0000121, PMID: 28368176 PMC5624819

[79] Linacre JM. Optimizing rating scale category effectiveness. J Appl Meas. 3:85–106. PMID: 11997586

[80] Choi SW Gibbons LE Crane PK. Lordif: an R package for detecting differential item functioning using iterative hybrid ordinal logistic regression/item response theory and Monte Carlo simulations. J Stat Softw. (2011) 39:1–30. doi: 10.18637/jss.v039.i08, PMID: 21572908 PMC3093114

[81] Crane PK Gibbons LE Jolley L van Belle G. Differential item functioning analysis with ordinal logistic regression techniques: DIF detect and difwithpar. Med Care. (2006) 44:S115–23. doi: 10.1097/01.mlr.0000245183.28384.ed 17060818

[82] Zumbo BD. A handbook on the theory and methods of differential item functioning (DIF): Logistic regression modeling as a unitary framework for binary and Likert-type (ordinal) item scores. Ottawa, Canada: Directorate of Human Resources Research and Evaluation, Department of National Defense (1999).

[83] Kim S-H Cohen AS Alagoz C Kim S. DIF detection and effect size measures for Polytomously scored items. J Educ Meas. (2007) 44:93–116. doi: 10.1111/j.1745-3984.2007.00029.x

[84] Felt JM Castaneda R Tiemensma J Depaoli S. Using person fit statistics to detect outliers in survey research. Front Psychol. (2017) 8:863. doi: 10.3389/fpsyg.2017.00863, PMID: 28603512 PMC5445123

[85] Drasgow F Levine MV William EA. Appropriateness measurement with polychotomous item response models and standardized indices. Br J Math Stat Psychol. (1985) 38:67–86. doi: 10.1111/j.2044-8317.1985.tb00817.x

[86] Ferrando PJ Chico E. Detecting dissimulation in personality test scores: A comparison between person-fit índices and detection scales. Educ Psychol Meas. (2001) 61:997–12. doi: 10.1177/2F00131640121971617

[87] Ferrando PJ Vigil-Colet A Lorenzo-Seva U. Practice person-fit assessment with linear FA model: new developments and a comparative study. Front Psychol. (2016) 7:1973. doi: 10.3389/fpsyg.2016.01973, PMID: 28082929 PMC5186803

[88] Reise SP Waller NG. Traitedness and the assessment of response pattern scalability. J Pers Soc Psychol. (1993) 65:143–1. doi: 10.1037/0022-3514.65.1.143

[89] Bock RD Aitkin M. Marginal maximum likelihood estimation of item parameters: application of an EM algorithm. Psychometrika. (1981) 46:443–9. doi: 10.1007/BF02293801

[90] Chapman R. Expected a posteriori scoring in PROMIS®. J Patient Rep Outc. (2022) 6:59. doi: 10.1186/s41687-022-00464-9, PMID: 35657454 PMC9166925

[91] Muraki E Engelhard G. Full-information item factor analysis: applications of EAP scores. Appl Psychol Meas. (1985) 9:417–16. doi: 10.1177/014662168500900411

[92] Calderwood C Bennett AA Gabriel AS Trougakos JP Dahling JJ. Too anxious to help? Off-job affective rumination as a linking mechanism between work anxiety and helping. J Occup Organ Psychol. (2018) 91:681–7. doi: 10.1111/joop.12220

[93] Ietsugu T Crane C Hackmann A Brennan K Gross M Crane RS. Gradually getting better: trajectories of change in rumination and anxious worry in mindfulness-based cognitive therapy for prevention of relapse to recurrent depression. Mindfulness. (2015) 6:1088–94. doi: 10.1007/s12671-014-0358-3

[94] Ximénez C. A Monte Carlo study of recovery of weak factor loadings in confirmatory factor analysis. Struct Equ Model Multidiscip J. (2006) 13:587–4. doi: 10.1207/s15328007sem1304_5

[95] Sijtsma K van der Ark LA. A tutorial on how to do a Mokken scale analysis on your test and questionnaire data. Br J Math Stat Psychol. (2017) 70:137–8. doi: 10.1111/bmsp.12078, PMID: 27958642

[96] Billiet JB McClendon MJ. Modeling acquiescence in measurement models for two balanced sets of items. Struct Equ Model Multidiscip J. (2000) 7:608–8. doi: 10.1207/S15328007SEM0704_5

[97] Maydeu-Olivares A Coffman DL. Random intercept item factor analysis. Psychol Methods. (2006) 11:344–2. doi: 10.1037/1082-989X.11.4.344, PMID: 17154751

[98] Cronbach L. Coefficient alpha and the internal structure of tests. Psychometrika. (1951) 16:297–4. doi: 10.1007/BF02310555

[99] Kroenke K Spitzer RL Williams JB. Anxiety disorders in primary care: prevalence, impairment, comorbidity, and detection. Ann Intern Med. (2007) 146:317–5. doi: 10.7326/0003-4819-146-5-200703060-00004, PMID: 17339617

[100] Nunnally J Bernstein I. Psychometric theory. 3rd ed. New York: McGraw-Hill.

[101] Cropley M Zijlstra FRH. Work and rumination In: Langan-Fox J Cooper CL, editors. Handbook of stress in the occupations. Cheltenham: Edward Elgar Publishing (2011)

[102] Hobfoll SE Shirom A. Conservation of resources theory: applications to stress and management in the workplace In: Golembiewski RT, editor. Handbook of organizational behavior. New York: Marcel Dekker (2001)

[103] Neveu J-P. Jailed resources: conservation of resources theory as applied to burnout among prison guards. J Organ Behav. (2007) 28:21–42. doi: 10.1002/job.393

[104] Payne N Kinman G. Job demands, resources and work-related well-being in UK firefighters. Occup Med. (2019) 69:604–9. doi: 10.1093/occmed/kqz167, PMID: 31925427

[105] Greden JF Garcia-Tosi R Harrington AW. Healthy minds at work: challenges and strategies for business In: Riba MB Parikh SV Greden JF, editors. Mental health in the workplace: Strategies and tools to optimize outcomes. Switzerland: Springer (2019)

[106] Taubman DS Velyvis V Parikh SV. Assessment and treatment of mood and anxiety disorders in the workplace In: Riba MB Parikh SV Greden JF, editors. Mental health in the workplace: Strategies and tools to optimize outcomes. Cham: Springer

[107] Brondino M Pasini M da Silva SCA. Development and validation of an integrated organizational safety climate questionnaire with multilevel confirmatory factor analysis. Qual Quant. (2013) 47:2191–23. doi: 10.1007/s11135-011-9651-6

